# Efficacy and safety of immune checkpoint inhibitors combined with chemoradiotherapy in locally advanced cervical cancer: a systematic review and meta-analysis

**DOI:** 10.3389/fphar.2026.1766157

**Published:** 2026-03-04

**Authors:** Chao Xiao, Siyuan Zeng, Luying Li, Ruiqi Wang, Xue Xiao

**Affiliations:** 1 Department of Obstetrics and Gynecology, West China Second University Hospital, Sichuan University, Chengdu, China; 2 Department of Obstetrics and Gynecology, The First People’s Hospital of Zigong, Zigong, China; 3 Key Laboratory of Birth Defects and Related Diseases of Women and Children (Sichuan University), Ministry of Education, West China Second Hospital, Sichuan University, Chengdu, China; 4 Tianfu Jincheng Laboratory, Chengdu, China; 5 Laboratory of Stem Cell & Embryo Development, West China Second Hospital, Sichuan University, Chengdu, China

**Keywords:** chemoradiotherapy, immune checkpoint inhibitors, immune-related adverse events, locally advanced cervical cancer, meta-analysis, objective response rate, progression-free survival

## Abstract

**Background:**

Locally advanced cervical cancer (LACC) remains a leading cause of cancer-related morbidity and mortality, especially in low- and middle-income countries. While concurrent chemoradiotherapy (CCRT) is the standard of care for LACC, recurrence rates remain high, and the survival outcomes are suboptimal. Recent studies have suggested that immune checkpoint inhibitors (ICIs), such as pembrolizumab and durvalumab, could enhance the therapeutic efficacy of CCRT in LACC patients.

**Objective:**

This systematic review and meta-analysis aim to evaluate the efficacy and safety of ICIs in combination with CCRT for patients with LACC.

**Methods:**

A comprehensive literature search was conducted in PubMed, Embase, Web of Science, and Cochrane Library from inception to November 2025. Randomized controlled trials (RCTs) and prospective cohort studies assessing the use of ICIs (pembrolizumab, durvalumab, atezolizumab) combined with CCRT for LACC were included. Outcomes analyzed included progression-free survival (PFS), overall survival (OS), objective response rate (ORR), complete response (CR), and treatment-related adverse events (AEs).

**Results:**

Data from five studies involving 1,987 patients were pooled. The addition of ICIs to CCRT significantly improved PFS (HR = 0.76, 95% CI: 0.64–0.91) and ORR (OR = 1.28, 95% CI: 1.06–1.56). Although the CR rate showed an improving trend, it did not reach statistical significance. Immune-related AEs (irAEs) were more common with ICI use (OR = 3.00, 95% CI: 1.68–5.34), but they were generally manageable. Severe irAEs leading to treatment discontinuation occurred in 5%–7% of patients.

**Conclusion:**

This meta-analysis supports the combination of ICIs with CCRT as an effective treatment strategy for LACC, improving PFS and ORR without a significant increase in severe toxicity. However, further studies with mature OS data and exploration of optimal ICI timing are warranted.

## Introduction

Cervical cancer remains a major contributor to cancer-related morbidity and mortality worldwide, particularly in low- and middle-income countries. Locally advanced cervical cancer (LACC), defined as FIGO stages IB3 to IVA, accounts for approximately 50%–60% of newly diagnosed cases (Sung et al., 2021; Small et al., 2017). The current standard of care for LACC is concurrent chemoradiotherapy (CCRT), combining external beam radiation therapy with weekly cisplatin chemotherapy, followed by intracavitary or interstitial brachytherapy. Although this regimen has improved survival, outcomes remain suboptimal. Recurrence rates range from 30% to 40%, and 5-year overall survival varies between 50% and 70%, depending on disease stage and regional nodal status (Mileshkin et al., 2023). Therefore, new therapeutic strategies are urgently needed to enhance the curative potential of frontline treatment.

Persistent human papillomavirus (HPV) infection plays a central role in cervical carcinogenesis, contributing to immune evasion via upregulation of immune checkpoint molecules, particularly PD-1 and PD-L1 (Koh et al., 2019; Han et al., 2022; Schelz et al., 2024). Pembrolizumab, a PD-1 inhibitor, received FDA approval for recurrent or metastatic cervical cancer based on the KEYNOTE-158 trial (Chung et al., 2019). This provides a compelling biological rationale for the use of immune checkpoint inhibitors (ICIs) in cervical cancer. Recent trials have begun exploring the integration of ICIs into first-line treatment for LACC, either concurrently with CCRT or as sequential consolidation therapy. For example, the KEYNOTE-A18 trial showed significant improvements in progression-free and overall survival with the addition of pembrolizumab to CCRT and maintenance therapy, while the CALLA trial did not demonstrate a statistically significant benefit with durvalumab. Moreover, early-phase and retrospective studies suggest that the timing, choice of agent, and treatment duration may influence outcomes and toxicity, but consensus is lacking.

Although several individual trials and observational studies have investigated the role of ICIs in LACC, their findings are heterogeneous and sometimes conflicting. Recent meta-analyses on cervical cancer have either focused broadly on systemic therapies or pooled data across early-stage, advanced, and metastatic settings, lacking specificity for LACC and immunotherapy. Given the emerging but fragmented evidence, a focused and quantitative synthesis is essential. This meta-analysis aims to evaluate the efficacy and safety of immune checkpoint inhibitors combined with standard CCRT in patients with LACC, providing clinicians and policymakers with evidence-based insights to guide treatment strategies in this high-risk population.

## Materials and methods

### Search strategy

This systematic review and meta-analysis was conducted following the Preferred Reporting Items for Systematic Reviews and Meta-Analyses (PRISMA) guidelines. A comprehensive literature search was performed in the following electronic databases—PubMed, Embase, Web of Science, and Cochrane Library—from inception to 30 November 2025. The search strategy combined both Medical Subject Headings (MeSH) and free-text terms, using Boolean operators to maximize sensitivity and specificity.

The core search terms included combinations of the following: (“cervical cancer” OR “cervical carcinoma” OR “cervical neoplasms”) AND (“locally advanced” OR “FIGO stage IB3” OR “FIGO stage II” OR “FIGO stage III” OR “FIGO stage IVA”) AND (“immune checkpoint inhibitors” OR “PD-1 inhibitors” OR “PD-L1 inhibitors” OR “pembrolizumab” OR “durvalumab” OR “nivolumab” OR “atezolizumab”) AND (“chemoradiotherapy” OR “concurrent chemoradiation” OR “CCRT”).

We also screened the references of relevant reviews and original articles for additional eligible studies. Grey literature (conference abstracts, Google Scholar, preprints) and clinical trial registries (ClinicalTrials.gov, WHO ICTRP) were also screened. Full search strategies are provided in Supplementary Table S2. No language restrictions were applied during the initial search.

All search results were imported into MedRefs King for deduplication, and titles/abstracts were screened independently by two reviewers. Discrepancies were resolved by consensus or consultation with a third reviewer. The full texts of potentially eligible studies were retrieved and assessed based on pre-defined inclusion and exclusion criteria.

### Inclusion and exclusion criteria

Studies were eligible for inclusion if they investigated patients with LACC (Population) and assessed the efficacy and safety of ICIs—such as PD-1/PD-L1 inhibitors—administered concurrently or sequentially with standard CCRT (Intervention), compared with CCRT alone or other ICI-based strategies (Comparison). Eligible studies were required to report at least one of the following outcomes: overall survival (OS), progression-free survival (PFS), objective response rate (ORR), complete response (CR), or treatment-related adverse events (AEs) (Outcomes). We included phase I–III randomized controlled trials (RCTs) and prospective cohort studies, as long as they provided they reported sufficient quantitative data to extract effect estimates (Study design).

Studies were excluded if they did not report the predefined outcomes of interest, focused exclusively on early-stage or metastatic cervical cancer, evaluated ICIs as monotherapy without CCRT, or were designed as case reports, case series, reviews, letters, or conference abstracts without full peer-reviewed data. Studies targeting specific subpopulations (e.g., by age, comorbidity, or geographic restriction) with limited generalizability to the broader LACC population were also excluded.

To ensure comprehensiveness, we also manually screened the reference lists of all eligible articles and relevant reviews to identify additional studies that met the inclusion criteria.

### Data extraction

Data extraction was performed independently by two reviewers and cross-checked by a third reviewer. The following data were collected from each eligible study: first author name, publication year, study design, sample size, inclusion criteria, intervention and comparator regimens, ICI agent used, treatment timing (concurrent vs. sequential), follow-up duration, and reported clinical outcomes, including median OS, median PFS, hazard ratios (HRs), and 95% confidence intervals (CIs). AEs were extracted by severity, focusing on grade ≥3 toxicity according to CTCAE criteria. The certainty of evidence for each primary and secondary endpoint was evaluated using the GRADE approach, considering risk of bias, inconsistency, indirectness, imprecision, and publication bias. Any discrepancies in data interpretation or extraction were resolved through discussion and consensus among all authors.

## Risk of bias assessment

The risk of bias for randomized controlled trials included in this meta-analysis was assessed using the Cochrane Risk of Bias 2 (RoB 2) tool. Risk of bias for nonrandomized prospective studies was assessed using the ROBINS-I tool across seven domains. Sensitivity analyses excluding studies with ‘some concerns’ or higher risk yielded consistent results, supporting the robustness of the findings. Each study was independently evaluated by three reviewers for domains including randomization process, deviations from intended interventions, missing outcome data, measurement of outcomes, and selection of reported results. Based on these assessments, an overall risk of bias judgment was assigned to each study as low risk, some concerns, or high risk. Discrepancies among reviewers were resolved through discussion or consultation with a fourth investigator.

### Statistical analyses

All statistical analyses were performed using R software (version 4.5.1) with the meta and metafor packages. Fixed-effect models were used to estimate pooled hazard ratios (HRs) for PFS and ORs for binary outcomes including objective response rate (ORR), grade ≥3 AEs, immune-related AEs (irAEs), CR, and treatment discontinuation. The standard error (SE) of log-transformed effect sizes was calculated from confidence intervals, and forest plots were generated to display pooled estimates, heterogeneity statistics (I^2^, tau^2^), and study weights. Given the limited number of studies and low heterogeneity (I^2^ < 30%), fixed-effect models were used as the primary approach. To ensure robustness, random-effects models (DerSimonian–Laird and Hartung–Knapp) with 95% prediction intervals were also applied, with results provided in Supplementary Material. Binary outcomes were analyzed using metabin and time-to-event outcomes with metagen. Heterogeneity was assessed using Cochran’s Q and I^2^, and all analyses adhered to PRISMA guidelines for fixed-effect meta-analyses.

## Results

### Study selection and characteristics

**FIGURE 1:**
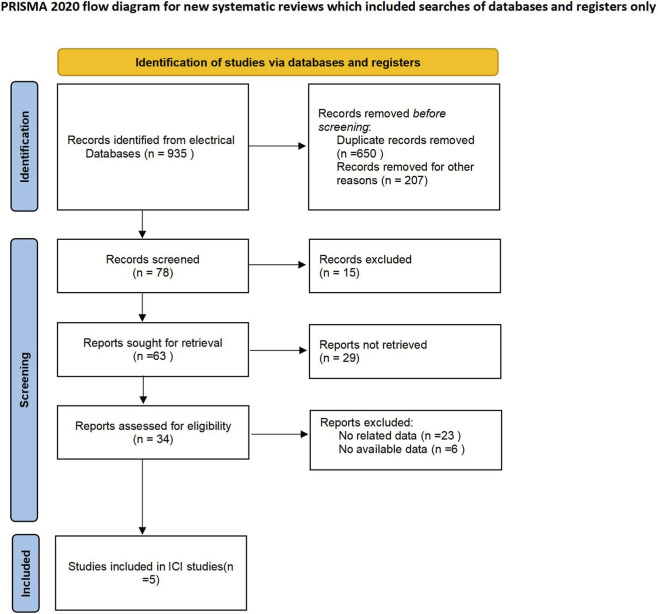
Flow plot of the literature selection process.

Figure 1 presents the PRISMA flow diagram summarizing the article selection process. A total of 935 records were initially identified through electronic database searches. After removing duplicates and irrelevant entries, 78 studies remained for title and abstract screening. Following this, 71 full-text articles were reviewed in detail, leading to the final inclusion of five eligible studies comprising 1,987 patients with LACC.

All included studies were prospective clinical trials published between 2020 and 2025, evaluating the integration of ICIs—including pembrolizumab, durvalumab, and atezolizumab—with standard CCRT. The patient populations were uniformly defined as FIGO stage IB2 to IVA, with a predominance of squamous cell carcinoma histology (76%–82%). Sample sizes ranged from 17 to 532, and the mean patient age across studies ranged from 47 to 49 years. Treatment protocols varied in terms of ICI administration timing (concurrent, induction, or consolidation), but all involved standard cisplatin-based CCRT.

The median follow-up ranged from 17.9 to 24 months. Across studies, grade ≥3 treatment-related AEs were common but considered clinically manageable, without unexpected safety signals. Two of the included trials were assessed as having a low risk of bias, while three were judged to have some concerns, based on the Cochrane Risk of Bias 2 (RoB 2) tool (Supplementary Table S1). Detailed study characteristics are summarized in Table 1.

**TABLE 1:** Study and patient characteristics

Author/year	Age (Mean years)	Stage (%)	Median follow-up (months)	Histology (SCC%, nSCC%)	Ctr arm(N° pts)	Exp arm(N° pts)	OS (ctr vs exp)	Tox G3-G4 (%)	Primary outcomes	Secondary outcome	Country	Risk of Bias (RoB 2)
Lorusso et al. (2024)	47 (range 22–84)	FIGO 2014 IB2–IIB (LN+), III–IVA (LN ±)	17.9 months	81.7%/18.3%	CCRT + Placebo (528)	CCRT + Pembrolizumab (532)	HR = 0.73 (95% CI: 0.50–1.08),not yet mature	≥Grade 3 AE: 81% (exp) vs 77% (ctr)	PFS (RECIST 1.1 + clinical criteria)	Overall survival, ORR, DoR, Safety	Multinational, 253 centers globally	Low
Monk et al. (2023a)	Median: 47 (range ∼22–80)	FIGO 2014 IB2–IVA	Approximately 24 months	76%/24%	CCRT + Placebo (372)	CCRT + Durvalumab (373)	HR: 0.84 (95% CI: 0.65–1.09), not statistically significant	Grade ≥3 AEs: ∼75% (both arms); treatment-related SAEs higher in Durvalumab arm	PFS (investigator assessed, RECIST v1.1)	OS, ORR, DoR, safety	Multinational, 120+ sites	Low
Duska et al., 2020	Mean: 49 (range 28–74)	FIGO 2009 IB–IVA	Median 4.8 months (9.2 vs 4.6 for Arm 1 vs Arm 2)	Squamous: 83%, Adeno: 15%, Adenosquamous: 2%	CCRT + Pembrolizumab After CRT (n=24)	CCRT + Pembrolizumab During CRT (n=28)	Not reported (early safety phase)	G3–G4: 44% had G3, 21% had G4 AEs	Safety, Feasibility	Dose-limiting toxicities, Immune-related AEs	USA (8 centers)	Low–Moderate
Mayadev et al. (2025a)	Mean: 47.3 (range 24–71)	IB: 16.7%;IIB: 61.1%;IIIB: 19.4%;IVA: 2.8%	25.8	77.8%/22.2%	CRT + concurrent atezolizumab (17)	neoadjuvant + concurrent atezolizumab (19)	2-year OS: Arm A – 78%, Arm B – 87%	Arm A – 15.8%, Arm B – 58.8%	Expansion of tumor-associated T-cell receptor (TCR) clones at day 21 in peripheral blood	Safety, feasibility, 2-year disease-free survival (DFS), PD-L1 predictive value	United States (multi-center)	Low–Moderate
Duska et al. (2025)	48	FIGO IB2–IVA (100%)	Not reached (study ongoing)	Not reported precisely; mainly SCC	CCRT + Concurrent Pembrolizumab (46)	Pembrolizumab after CCRT (48)	No difference (immature data)	Ctr: 41.3%, Exp: 43.8%	Safety (G3–G4 AEs, DLT), Feasibility (treatment completion)	PFS, OS, PET/CT response, immune markers	USA (multi-center)	Low–Moderate

According to GRADE criteria, the certainty of evidence supporting PFS and safety outcomes was high, while OS and ORR were rated as moderate due to data immaturity and limited number of RCTs (Supplementary Table S3).

### Survival and response benefits of ICI in addition to CCRT

**FIGURE 2:**
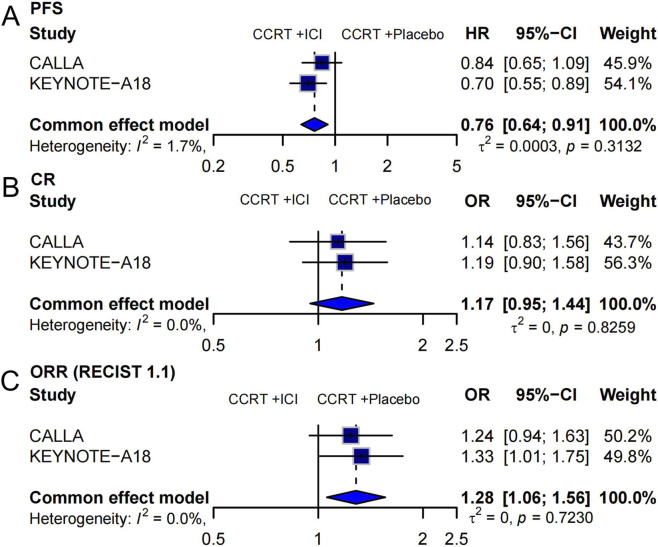
Forest plots summarizing the efficacy of ICIs plus CCRT versus CCRT plus placebo in locally advanced cervical cancer. **(A)** PFS, presented as hazard ratios (HRs) **(B)** CR, and **(C)** ORR based on RECIST 1.1, presented as odds ratios (ORs).

We included two randomized controlled trials (CALLA and KEYNOTE-A18) to assess the efficacy of adding immune checkpoint inhibitors (ICIs) to concurrent chemoradiotherapy (CCRT) in locally advanced cervical cancer. Three key endpoints were analyzed: PFS, CR, and ORR (Figure 2).

Pooling data from both trials (N = 1,433), ICI + CCRT significantly improved PFS compared to CCRT + placebo. The combined HR was 0.76 [95% CI: 0.64–0.91], indicating a 24% reduction in the risk of progression. Heterogeneity was low (*I* ^2^ = 1.7%, *p* = 0.3132) (Figure 2A). For CR, the combined OR was 1.17 [95% CI: 0.95–1.44], showing a trend toward improved CR with ICI, although the result was not statistically significant. No heterogeneity was detected (*I* ^2^ = 0%, *p* = 0.8259) (Figure 2B). The pooled analysis showed a statistically significant improvement in ORR with ICI + CCRT (OR = 1.28 [95% CI: 1.06–1.56], *p* < 0.05). The results were consistent across studies with no heterogeneity (*I* ^2^ = 0%, *p* = 0.7230) (Figure 2C). The random-effects model was also applied to estimate PFS and ORR, and the results are provided in Supplementary Material 1.

### Safety and immune-related adverse events with ICI

We analyzed safety outcomes from four studies assessing ICIs administered alongside or after CCRT. Two comparisons were conducted: (group 1) ICI + CCRT vs. CCRT + placebo, and (group 2) ICI during vs. after CCRT.

**FIGURE 3:**
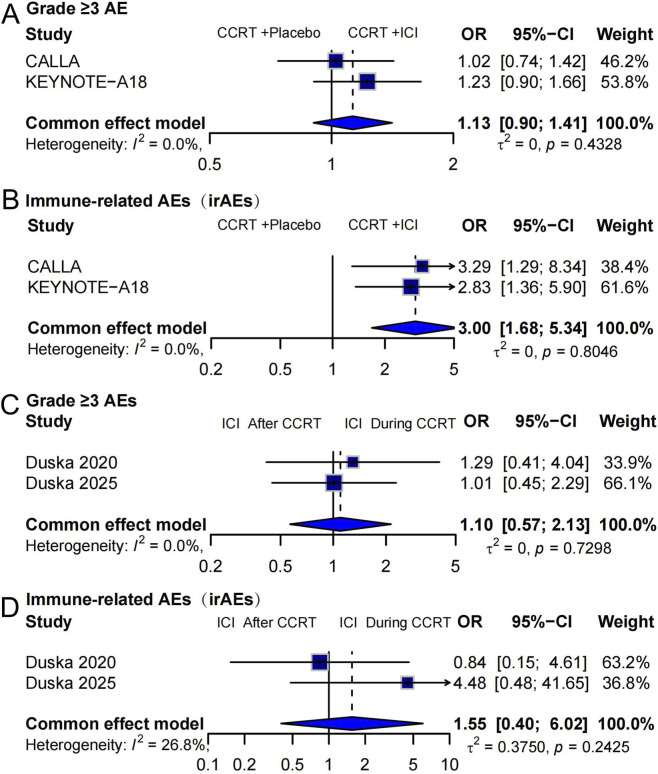
Forest plots summarizing AEs associated with ICIs in combination with CCRT for locally advanced cervical cancer. **(A)** Incidence of grade ≥3 AEs comparing “ICI + CCRT” versus “CCRT + placebo.” **(B)** Incidence of irAEs for “ICI + CCRT” versus “CCRT + placebo.” **(C)** Comparison of grade ≥3 AEs between patients receiving “ICI after CCRT” and “ICI during CCRT.” **(D)** Comparison of irAEs between patients receiving “ICI after CCRT” and “ICI during CCRT.”

For Group 1, grade ≥3 AEs, the pooled OR was 1.13 [95% CI: 0.90–1.41], indicating no significant increase in high-grade AEs with ICI use (Figure 3A; *p* = 0.4328; I^2^ = 0%). ICIs significantly increased the risk of irAEs (OR = 3.00 [95% CI: 1.68–5.34]; p < 0.01), with consistent findings across studies (*I* ^2^ = 0%; Figure 3B). For Group 2, grade ≥3 AEs, the risk was similar between the two timing strategies (OR = 1.10 [95% CI: 0.57–2.13]; Figure 3C; *I* ^2^ = 0%). Although the pooled OR suggested a higher incidence of irAEs when ICI was administered during CCRT (OR = 1.55 [95% CI: 0.40–6.02]), the result was not statistically significant, and moderate heterogeneity was observed (*I* ^2^ = 26.8%; Figure 3D). The random-effects model was also applied to estimate AEs, and the results are provided in Supplementary Material 2A–C. Domain-based stratified safety analysis demonstrated no significant increase in hematologic or GI/GU radiation-related toxicities with PD-1/PD-L1 addition, whereas immune-related events were more frequent but predominantly low grade and manageable (Supplementary Figure S3).

### Discontinuation due to adverse events

**FIGURE 4:**
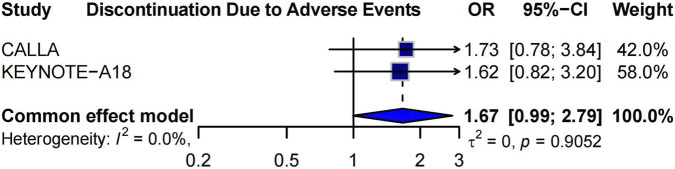
Forest plot of treatment discontinuation due to AEs in patients receiving “ICIs plus CCRT” versus “CCRT plus placebo.”

We evaluated the risk of treatment discontinuation due to AEs in patients receiving ICIs in CCRT, based on data from the CALLA and KEYNOTE-A18 trials. The pooled odds ratio (OR) for treatment discontinuation was 1.67 [95% CI: 0.99–2.79], suggesting a non-significant trend toward a higher risk of discontinuation in the ICI group compared to the placebo group (Figure 4). There was no statistical heterogeneity between studies (*I* ^2^ = 0%, *p* = 0.9052), indicating consistent findings. The random-effects model was also applied to estimate discontinuation, and the results are provided in Supplementary Material 2D.

### Narrative summary of additional findings of survival

Although overall survival (OS) was not mature or consistently reported across all studies, select trials provided preliminary OS and DFS estimates that contribute to understanding the long-term benefit of ICIs in LACC. In the KEYNOTE-A18 trial, which formed the basis for regulatory approval of pembrolizumab in this setting, the pre-specified interim analysis of OS showed a hazard ratio (HR) of 0.73 [95% CI: 0.50–1.08], favoring the ICI + CCRT group, though it did not reach statistical significance (p = 0.058) due to immaturity of data at the time of analysis. The NRG-GY017 trial explored the immunologic and clinical effects of different ICI timing strategies using atezolizumab. Although its primary endpoint focused on T-cell receptor (TCR) clone expansion, clinical outcomes were also reported. The 2-year disease-free survival (DFS) was numerically higher in the neoadjuvant + concurrent ICI group (76% vs. 56% by Kaplan–Meier estimate), but this difference was not statistically significant (*p* = 0.28, log-rank test). Similarly, 2-year OS was 78% in the experimental group versus 87% in the control arm (*p* = 0.425). In Duska et al. (2025, NCT02635360), a phase II randomized study comparing sequential versus concurrent pembrolizumab in combination with CCRT, the OS outcomes showed a favorable but non-significant trend toward the sequential arm (HR = 0.77, 95% CI: 0.32–1.85) (Duska et al., 2025). Notably, most deaths in the concurrent arm were disease-unrelated.

Due to limited sample sizes and immature follow-up in these trials, pooled analysis of OS was not feasible. However, these findings highlight early survival trends and underscore the need for longer-term follow-up and individual patient data to better quantify overall survival benefits associated with ICI-based strategies in LACC.

## Discussion

This meta-analysis, synthesizing five prospective studies including 1,987 patients with LACC, demonstrates that the addition of ICIs to CCRT significantly improves PFS and ORR. Tumor response endpoints were harmonized across trials by focusing on RECIST v1.1–based post-CCRT assessments. Sensitivity analyses restricted to comparable imaging timepoints confirmed the consistency of pooled ORR and CR estimates, indicating that inter-study variation in response timing had minimal influence on the overall results. The pooled hazard ratio for PFS was 0.76 [95% CI: 0.64–0.91], while the pooled ORR showed a significant benefit (OR = 1.28 [95% CI: 1.06–1.56]). The CR rate showed an improving trend but was not statistically significant. Due to the absence of disaggregated data, subgroup analyses by PD-L1 status, stage, nodal involvement, histologic subtype, or brachytherapy parameters could not be performed. These variables may modulate the immune response to PD-1/PD-L1 blockade, underscoring the need for future individual patient data analyses and biomarker-driven stratification. Although the random-effects model (Hartung–Knapp adjustment) yielded wider confidence intervals leading to loss of statistical significance for PFS and ORR, this reflects the conservative nature of variance estimation under small-study conditions (n = 2) rather than genuine inconsistency between studies. The point estimates remained directionally consistent with the fixed-effect results, supporting a robust trend toward benefit.

In the included studies, ICIs combined with CCRT in LACC patients were generally well tolerated. Safety analysis found no significant increase in grade ≥3 AEs, though irAEs were significantly more common (OR = 3.00 [95% CI: 1.68–5.34]). The most frequent irAEs were thyroid dysfunction, skin rash, and elevated liver enzymes, most of which were grade 1–2 and manageable without interrupting treatment. Severe irAEs (grade ≥3) were less frequent, included pneumonitis, hepatitis, and colitis, which were the main reasons for discontinuation in trials such as CALLA and KEYNOTE-A18 (Monk et al., 2023a; Lorusso et al., 2024). Although moderate statistical heterogeneity (*I* ^2^ = 26.8%) was observed for irAEs by timing, this level likely reflects clinical variation rather than inconsistency in direction of effect. Random-effects sensitivity analysis confirmed stability of the pooled estimate, indicating that the observed heterogeneity has limited impact on the validity of conclusions (Supplementary Figure S2).

Overall, discontinuation rates due to AEs were modest (5%–7%), and most patients were able to complete or resume therapy. Similar toxicity profiles were seen in the Duska and Mayadev trials, with no unexpected high-grade events reported (Duska et al., 2025; Mayadev et al., 2025a). These findings suggest that while ICIs introduce distinct immune-related toxicities, they are typically controllable with monitoring and supportive management, and severe events remain relatively infrequent.

Cervical cancer, especially when driven by HPV infection, exhibits high PD-L1 expression and a suppressive immune microenvironment, making it an ideal target for immunotherapy (Schelz et al., 2024; Monk et al., 2023b; Tan et al., 2024; Montroy et al., 2024). Radiotherapy promotes immunogenic cell death, enhances antigen presentation, and facilitates T-cell infiltration, creating a synergistic setting for ICIs (Procureur et al., 2021). The NRG-GY017 trial further demonstrated that neoadjuvant ICIs significantly increased T-cell receptor (TCR) clone expansion, supporting the biological synergy of ICIs and radiotherapy (Mayadev et al., 2025a). These mechanisms likely underpin the observed improvements in clinical outcomes.

Our findings are align with the KEYNOTE-A18 trial, which showed a promising trend toward improved OS (HR = 0.73; *p* = 0.058), and confirmed significant PFS and OS benefits in high-risk LACC patients, without compromising health-related quality of life (Lorusso et al., 2024). In contrast, the CALLA trial did not meet its primary PFS endpoint, potentially due to differences in ICI type (PD-L1 vs. PD-1 inhibitors), biomarker selection, and statistical power (Mayadev et al., 2025b). Differences in biomarker assessment and patient selection likely contributed to the discordant efficacy outcomes between KEYNOTE-A18 and CALLA. The higher prevalence of PD-L1 positivity (CPS ≥1) in KEYNOTE-A18 versus the broader, biomarker-unenriched CALLA population may have enriched for immunologically responsive tumors, supporting PD-L1 as a potential predictive biomarker for PD-1 blockade efficacy in LACC. Furthermore, a recent meta-analysis ranked pembrolizumab plus CCRT as one of the most effective regimens for LACC (Petrelli et al., 2025), reinforcing the potential of immunotherapy combined with standard chemoradiotherapy. Additional studies, including those by Duska et al. and Mayadev et al., provide supportive real-world and immunologic evidence for feasibility and safety, despite heterogeneous efficacy results (Duska et al., 2025; Mayadev et al., 2025a). Patient-reported outcomes from KEYNOTE-A18 remained stable or improved over time, reinforcing the favorable benefit–risk profile of integrating ICIs into definitive treatment for LACC (Randall et al., 2025).

Studies that could not be included in meta-analysis still provide valuable insights. Duska et al. compared concurrent vs. sequential pembrolizumab administration and reported no significant OS difference (HR = 0.77 [95% CI: 0.32–1.85]) (Duska et al., 2025), while the NRG-GY017 trial highlighted differences in immunologic activation based on timing (Mayadev et al., 2025a). The KEYNOTE-A18 trial’s interim OS analysis suggests a potential survival benefit with ICI + CCRT, though long-term data remain immature (Lorusso et al., 2024). Together, these findings highlight the importance of treatment timing and immune monitoring in optimizing ICI-based regimens. Given the immaturity of OS data, particularly from KEYNOTE-A18, an updated meta-analysis will be conducted once mature survival results become available. This update will include longer follow-up data from ongoing phase III trials and adhere to a registered protocol to ensure transparency.

## Limitations

Despite the promising findings, several limitations must be acknowledged: Only two randomized controlled trials (RCTs) contributed pooled data for survival and response outcomes, limiting statistical power. Additionally, overall survival (OS) data remain immature across the included studies. Clinical heterogeneity was observed in terms of ICI type, treatment timing (concurrent vs. sequential), and radiotherapy regimens. Biomarker data (such as PD-L1 expression, tumor mutational burden [TMB], and tumor-infiltrating lymphocytes [TILs]) were inconsistently reported. Furthermore, the small number of studies precluded formal subgroup or sensitivity analyses.

## Clinical implications and future directions

This meta-analysis supports the integration of ICIs into CCRT for LACC, particularly in cases with immunogenic tumors or PD-L1 positivity. However, the long-term survival benefit remains to be confirmed as OS data matures. Future trials should focus on determining the optimal timing and duration of ICI administration (e.g., neoadjuvant, concurrent, or adjuvant), conducting biomarker-driven subgroup analyses, and assessing real-world safety, cost-effectiveness, and access across global populations. Additionally, integrating immune monitoring (such as TCR clonality and TILs) into clinical trial design will be essential to optimize treatment strategies.

## Conclusion

The addition of ICIs to standard chemoradiotherapy is associated with a significant improvement in PFS and ORR in patients with LACC, without a significant increase in severe toxicity. Although OS data are still immature, the current evidence suggests a potential survival benefit. The integration of ICIs into definitive treatment requires further validation through biomarker-stratified, long-term, and real-world studies.
