# Supplementary material for: Efficacy and safety of immune checkpoint inhibitors combined with chemoradiotherapy in locally advanced cervical cancer: a systematic review and meta-analysis
Source: Front Pharmacol. 2026 Mar 4;17:1766157. doi: 10.3389/fphar.2026.1766157 (PMC12996201; doi:10.3389/fphar.2026.1766157)
Supplement: Supplementary file 1 [file DataSheet2.pdf]

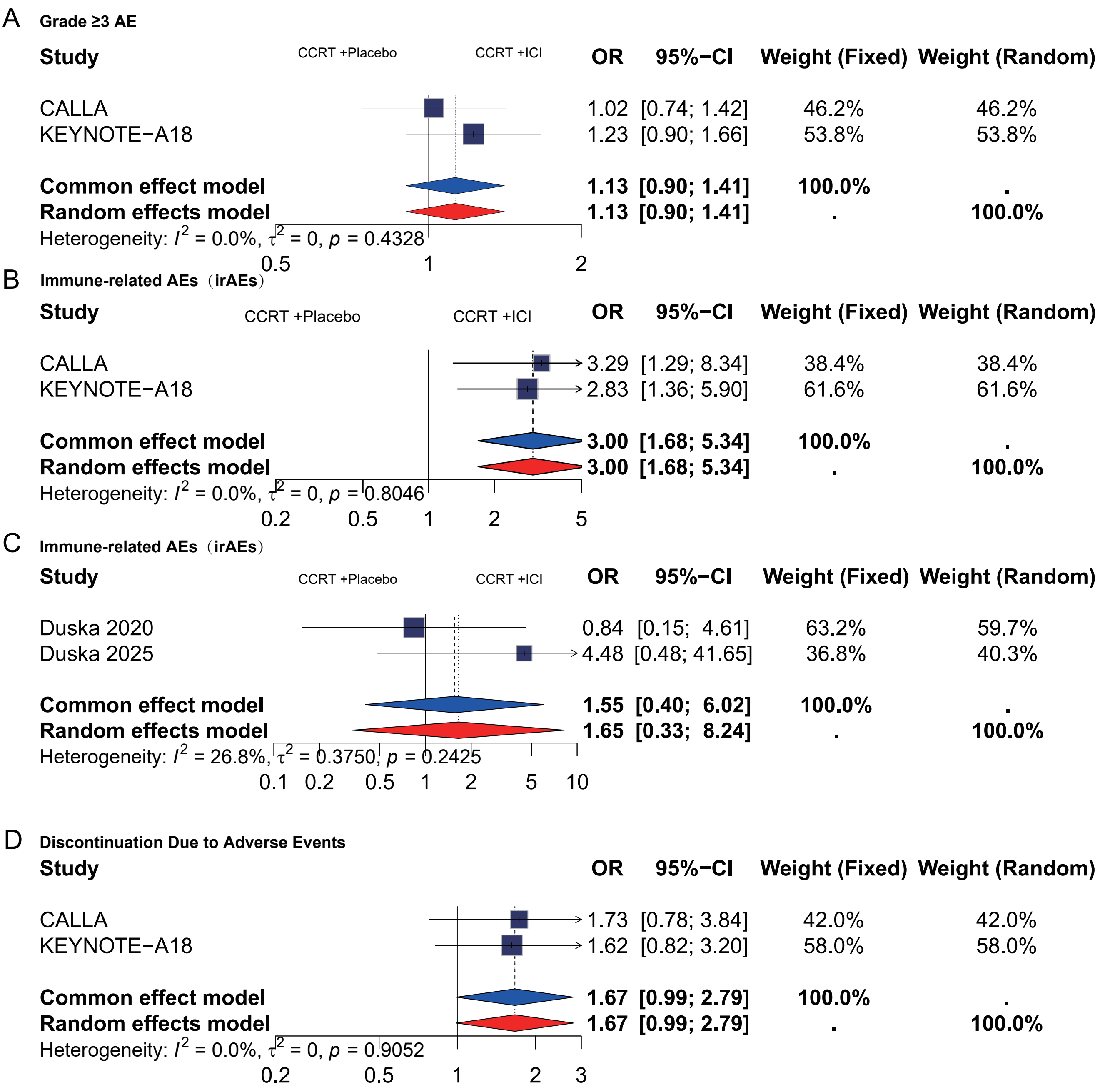
A
Grade ≥3 AE
Study
CCRT +Placebo
CCRT +ICI
OR
95%−CI
Weight (Fixed)
Weight (Random)
CALLA 1.02 [0.74; 1.42] 46.2% 46.2%
KEYNOTE−A18 1.23 [0.90; 1.66] 53.8% 53.8%
Common effect model 1.13 [0.90; 1.41] 100.0% .
Random effects model 1.13 [0.90; 1.41] . 100.0%
Heterogeneity: I^2 = 0.0%, τ^2 = 0, p = 0.4328
0.5
1
2
B
Immune-related AEs（irAEs）
Study
CCRT +Placebo
CCRT +ICI
OR
95%−CI
Weight (Fixed)
Weight (Random)
CALLA 3.29 [1.29; 8.34] 38.4% 38.4%
KEYNOTE−A18 2.83 [1.36; 5.90] 61.6% 61.6%
Common effect model 3.00 [1.68; 5.34] 100.0% .
Random effects model 3.00 [1.68; 5.34] . 100.0%
Heterogeneity: I^2 = 0.0%, τ^2 = 0, p = 0.8046
0.2
0.5
1
2
5
C
Immune-related AEs（irAEs）
Study
CCRT +Placebo
CCRT +ICI
OR
95%−CI
Weight (Fixed)
Weight (Random)
Duska 2020 0.84 [0.15; 4.61] 63.2% 59.7%
Duska 2025 4.48 [0.48; 41.65] 36.8% 40.3%
Common effect model 1.55 [0.40; 6.02] 100.0% .
Random effects model 1.65 [0.33; 8.24] . 100.0%
Heterogeneity: I^2 = 26.8%, τ^2 = 0.3750, p = 0.2425
0.1
0.2
0.5
1
2
5
10
D
Discontinuation Due to Adverse Events
Study
OR
95%−CI
Weight (Fixed)
Weight (Random)
CALLA 1.73 [0.78; 3.84] 42.0% 42.0%
KEYNOTE−A18 1.62 [0.82; 3.20] 58.0% 58.0%
Common effect model 1.67 [0.99; 2.79] 100.0% .
Random effects model 1.67 [0.99; 2.79] . 100.0%
Heterogeneity: I^2 = 0.0%, τ^2 = 0, p = 0.9052
0.2
0.5
1
2
3
