# Supplementary figures and images for: Efficacy and safety of immune checkpoint inhibitors combined with chemoradiotherapy in locally advanced cervical cancer: a systematic review and meta-analysis
Source: Front Pharmacol. 2026 Mar 4;17:1766157. doi: 10.3389/fphar.2026.1766157 (PMC12996201; doi:10.3389/fphar.2026.1766157)

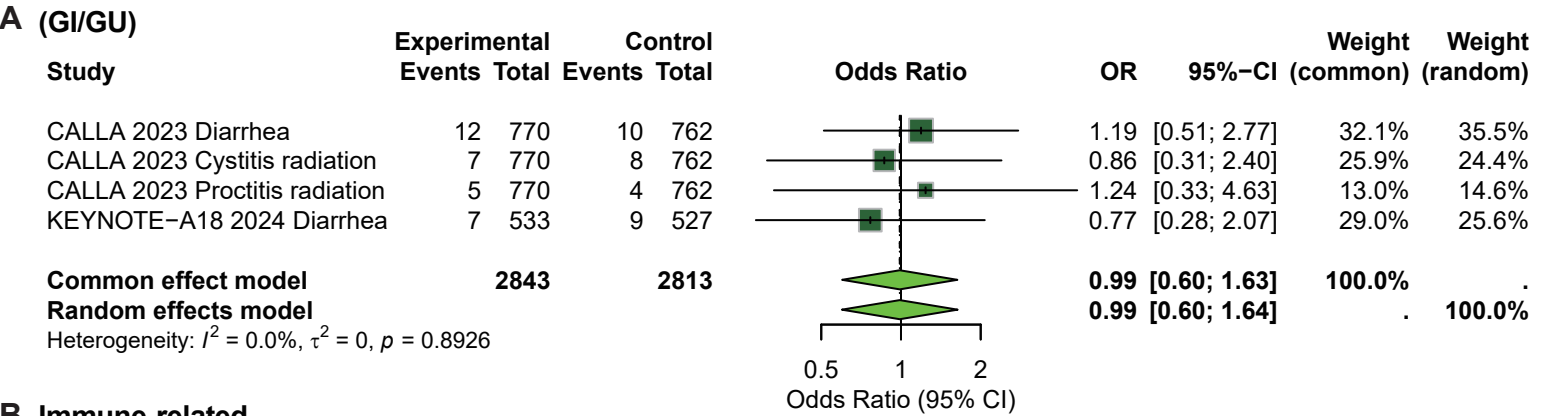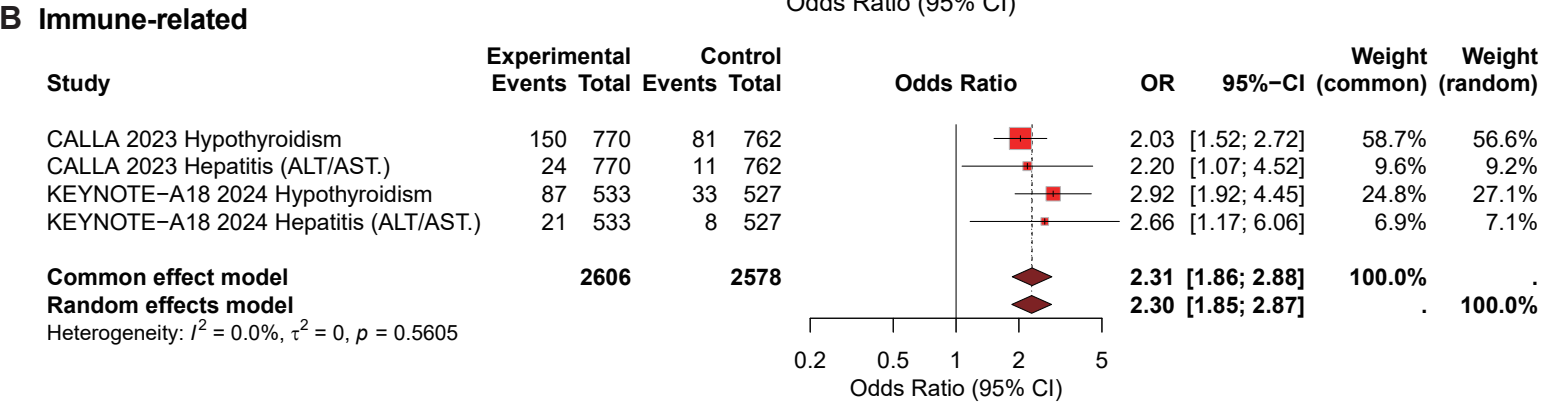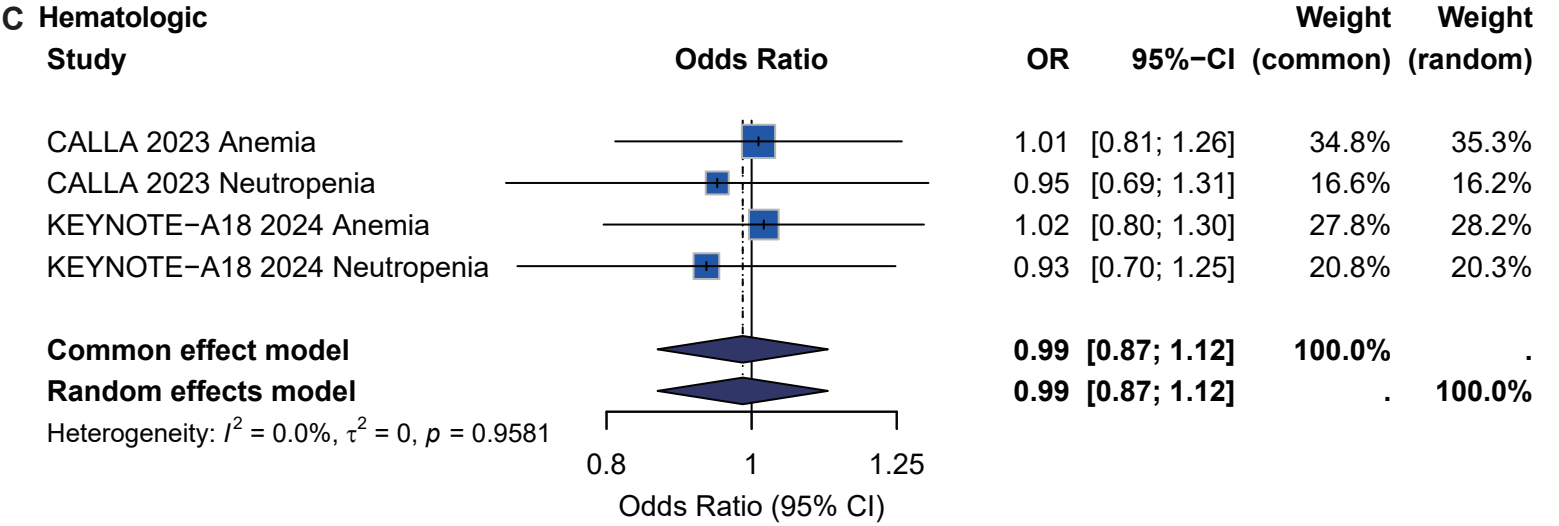

Supplement: Supplementary file 4 [file DataSheet3.pdf]

## A PFS

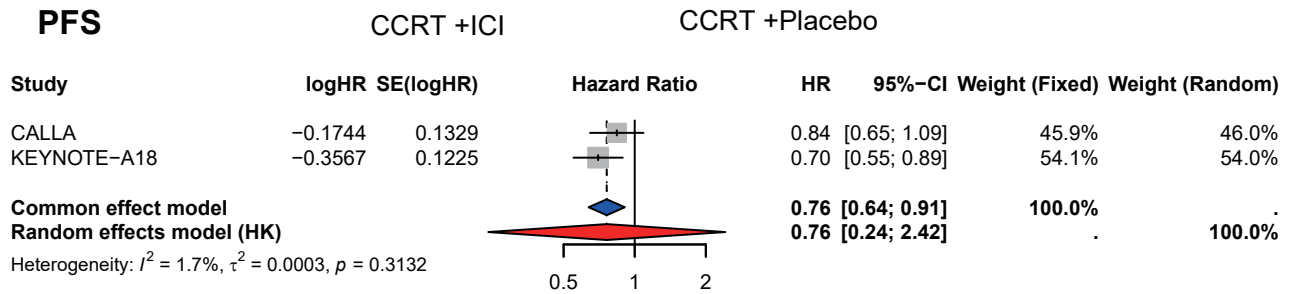

## B CR

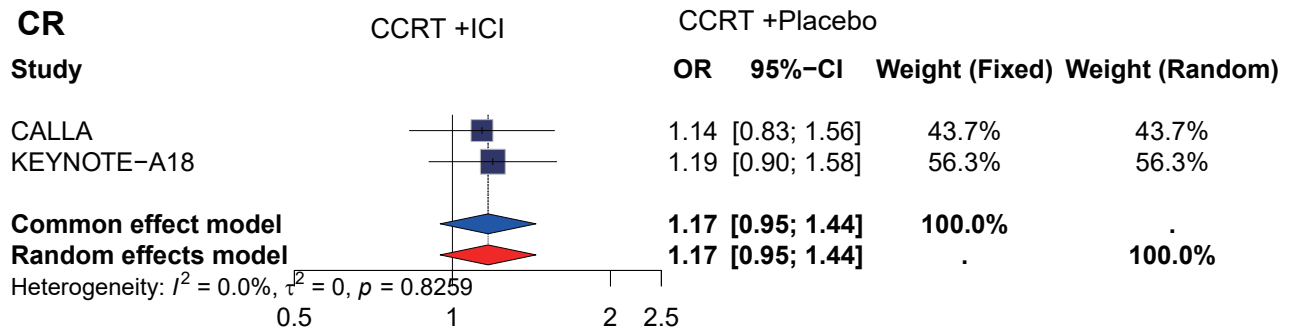

## C ORR (RECIST 1.1)

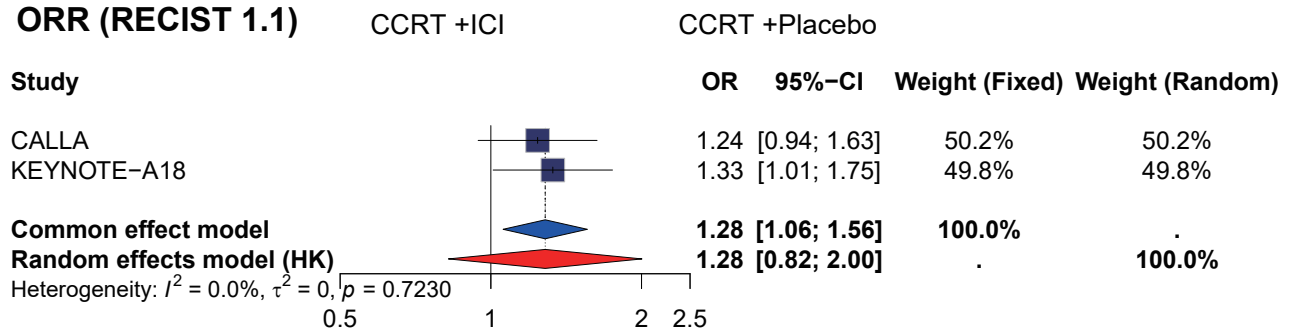

Supplement: Supplementary file 5 [file DataSheet1.pdf]
